# Supplementary material for: Pseudomonassin, a New Bioactive Ribosomally Synthesised and Post-Translationally Modified Peptide from Pseudomonas sp. SST3
Source: Microorganisms. 2023 Oct 15;11(10):2563. doi: 10.3390/microorganisms11102563 (PMC10609385; doi:10.3390/microorganisms11102563)
Supplement: Supplementary file 1 [file microorganisms-11-02563-s001.zip › microorganisms-2650276-supplementary (1)-done.pdf]

Pseudomonassin, a New Bioactive Ribosomally Synthesised and Post-Translationally Modified Peptide from *Pseudomonas* sp. SST3

Kevin Jace Miranda [1,2,*], Saif Jaber [3], Dana Atoum [3], Subha Arjunan [1], Rainer Ebel [1], Marcel Jaspars [1] and RuAngelie Edrada-Ebel [3]

[1] Marine Biodiscovery Centre, Department of Chemistry, University of Aberdeen, Meston Walk, Aberdeen AB24 3UE, UK; subha.arjunan@syngenta.com (S.A.); r.ebel@abdn.ac.uk (R.E.); m.jaspars@abdn.ac.uk (M.J.)
[2] College of Pharmacy and Graduate School, Adamson University, 900 San Marcelino Street, Ermita, Manila 1000, Philippines
[3] Strathclyde Institute of Pharmacy and Biomedical Sciences, University of Strathclyde, John Arbuthnott Building, 161 Cathedral Street, Glasgow G4 0RE, UK; saif.jaber@strath.ac.uk (S.J.); dana.atoum@hu.edu.jo (D.A.); ruangelie.edrada-ebel@strath.ac.uk (R.E.-E.)
* Correspondence: kevin.jace.miranda@adamson.edu.ph

Supplementary Information

Table of Contents

Page

Figure S1. Predicted structures of lasso peptide from RiPPMiner ……………………………………………………………..……...…3

Figure S2. HCD-MS/MS fragmentation of pseudomonocin showing all the *a*, *b* and *y* ions…………………………………………..……………………………………………………………..………....….4

Figure S3. Increasing energy spectra of pseudomonocin in HCD-MS/MS analysis………………………………………………... 5

Figure S4. Base peak of extracts showing the relative intensity of the target lasso peptide………..…………5

Table S1. Dereplicated compounds derived from Pseudomonas species...6

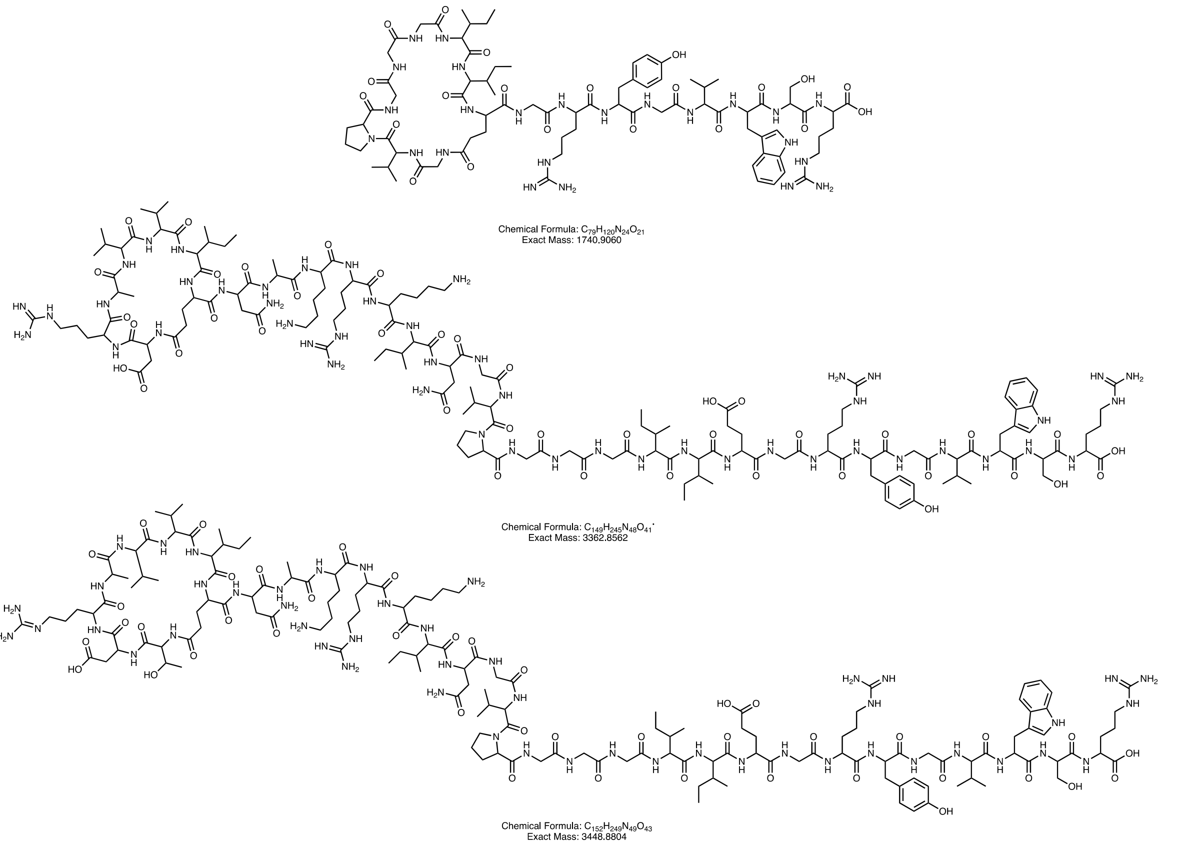

Figure S1. Predicted structures of lasso peptide from the gene cluster of *Pseudomonas* sp. SST3 from RiPPMiner.

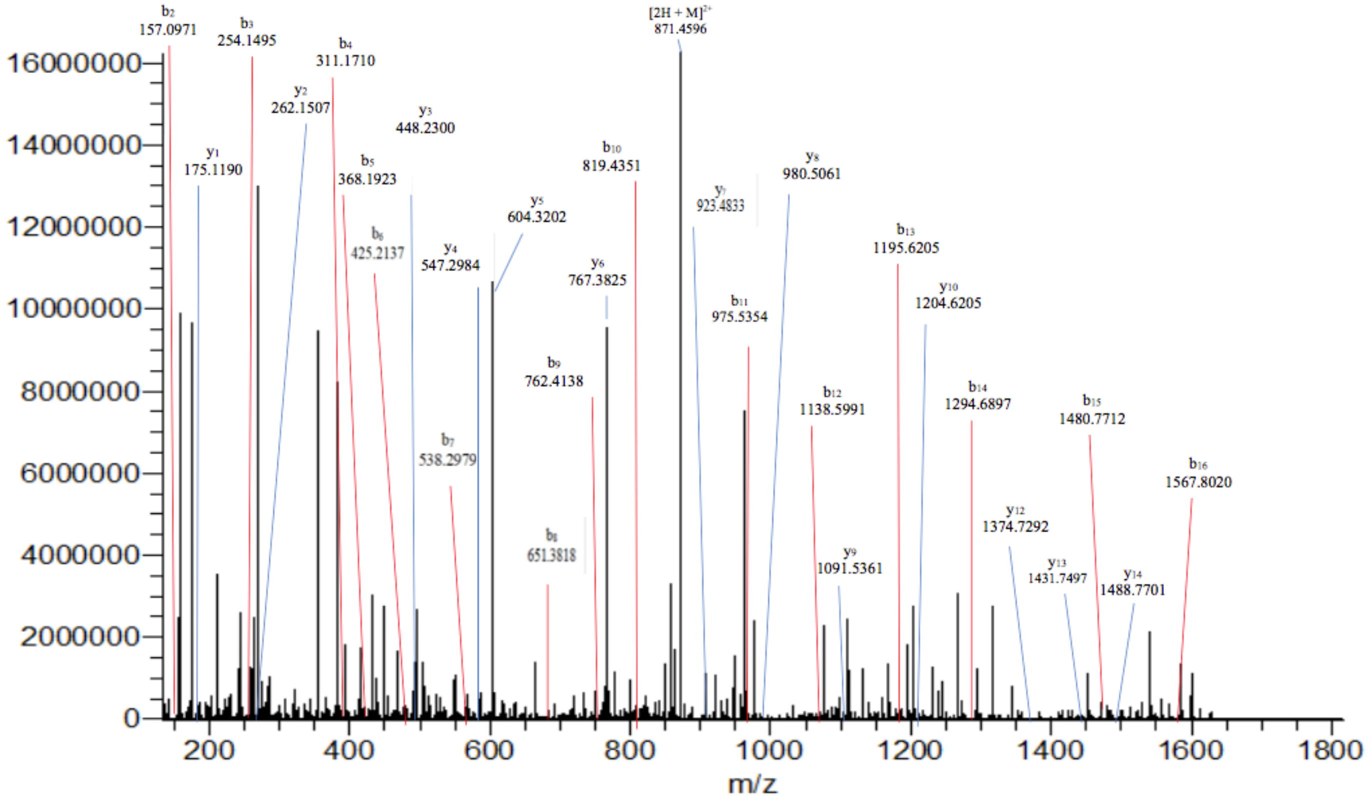

Figure S2. HCD-MS/MS fragmentation of pseudomonocin showing all the *a*, *b* and *y* ions.

20% NCE

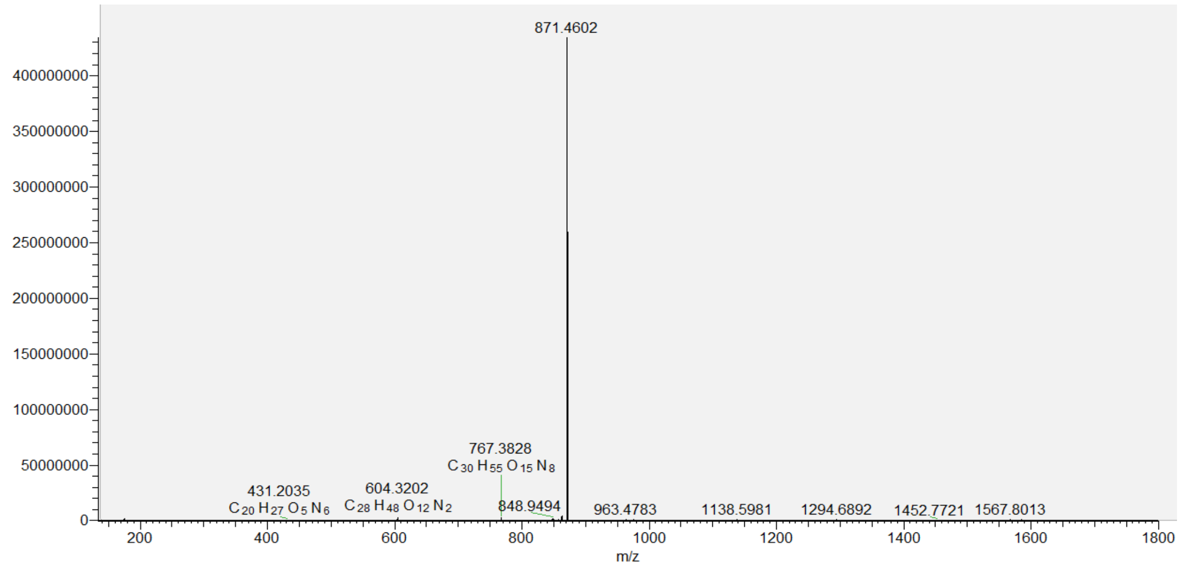

30% NCE

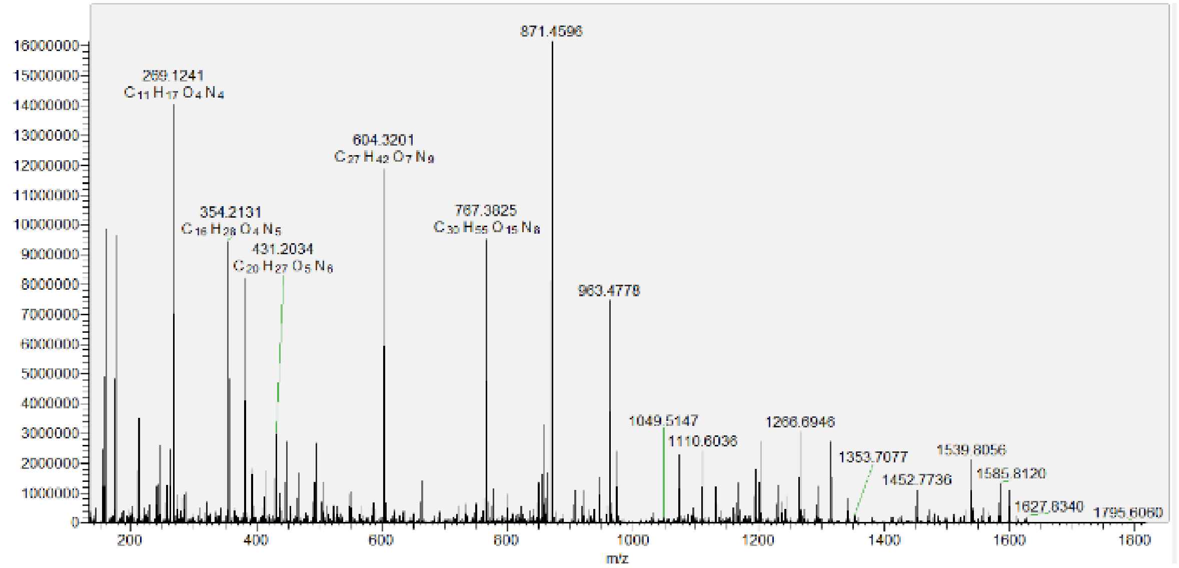

40% NCE

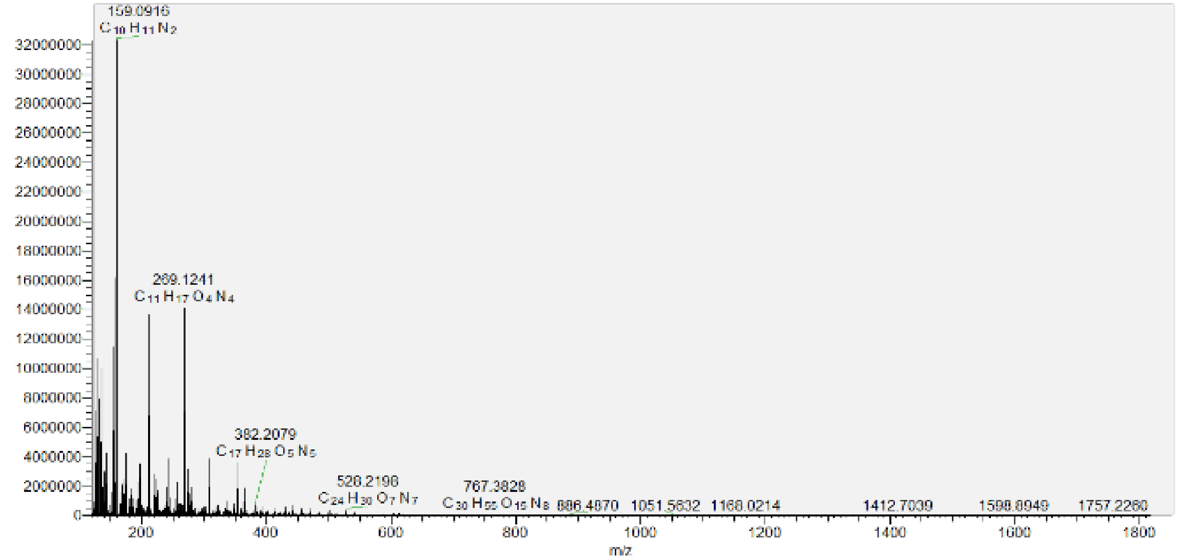

50% NCE

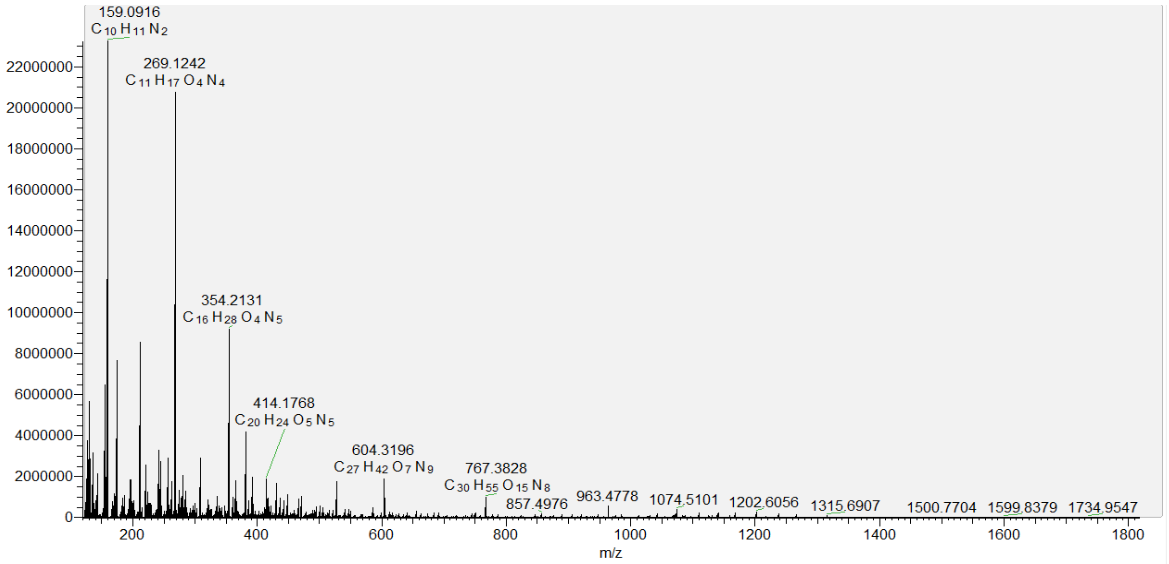

Figure S3. Increasing energy spectra of the lasso peptide in HCD-MS/MS analysis.

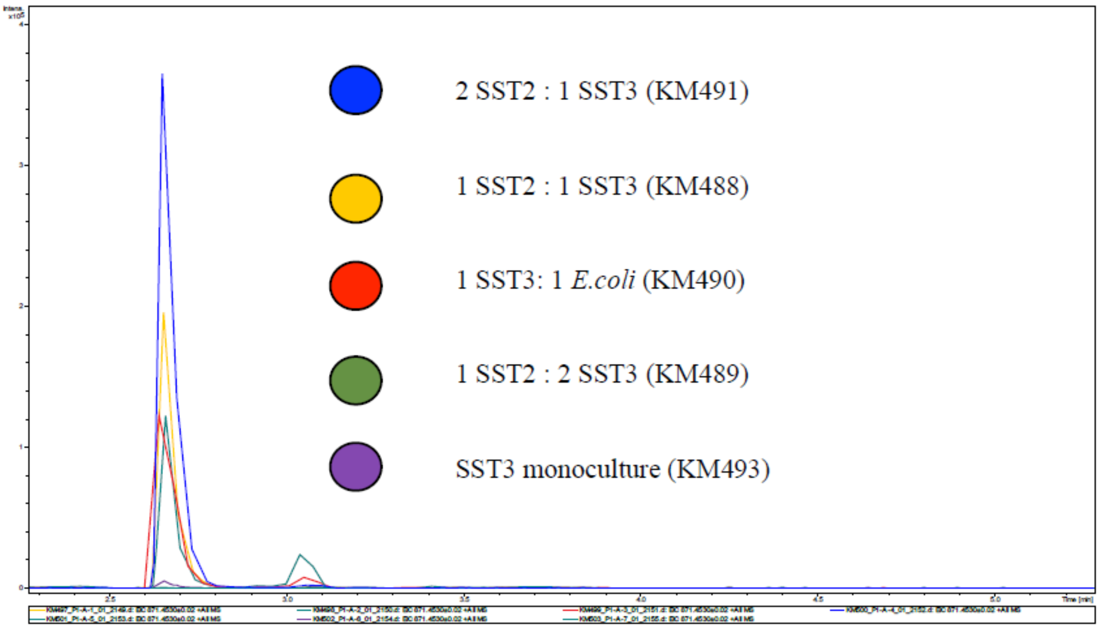

Figure S4. Base peak of extracts showing the relative intensity of the target lasso peptide.

Table S1. Dereplicated compounds derived from *Pseudomonas* species.

MZMine ID	RT (min)	*m/z*	MW (accuracy in ppm)	Formula prediction/RDB	Hits in DNP database	Source
**3305**	1.50	261.1226	260.1154 (-2.82)	$C_{14}H_{16}N_2O_3$ RDB = 8	Cyclo(prolyltyrosyl); (3S,8aS)-form	Isolated from the sponges *Jaspis digonoxea* and *Tedania anhelans* with a marine-derived *Pseudomonas aeruginosa*
**8898**	1.55	499.2896	498.2823 (-1.23)	$C_{26}H_{42}O_9$ RDB = 6	Mupirocin F2 **(4.9)**	Produced by the mupF mutant of *Pseudomonas fluorescens*
**9253**	1.67	480.2501	479.2428 (1.72)	$C_{27}H_{33}N_3O_5$ RDB = 13	Andrimide **(4.10)**	Produced by a bacterial symbiont from *Nilaparvata lugens*, a marine *Pseudomonas fluorescens* and an *Enterobacter* sp.
**3710**	1.72	454.2325	453.2252 (-2.57)	$C_{25}H_{31}N_3O_5$ RDB = 12	Moiramide B **(4.11)**	Metabolite of a marine *Pseudomonas fluorescens*
**3180**	1.73	489.2342	488.2269 (-0.38)	$C_{24}H_{32}N_4O_7$ RDB = 11	Cepaciacheli	Isolated from *Burkholderia cepacia* (*Pseudomonas cepacia*)
**3699**	1.79	245.1167	244.1094 (-2.08)	$C_{15}H_{16}O_3$ RDB = 8	1-(3,5-Dihydroxyphenyl)-2-(2-hydroxyphenyl)ethane; 3-methylether	Constituent of the bulbs of *Dioscorea batatus* (Chinese yam) infected with *Pseudomonas chichorii*
**4504**	1.79	310.1643	309.1570 (-2.02)	$C_{16}H_{23}NO_5$ RDB = 6	Coronafacic acid; L-Threonine amide	Produced by *Pseudomonas syringae*
**2652**	1.87	525.2694	524.2621 (-2.64)	$C_{28}H_{36}N_4O_6$ RDB = 13	Safracin A **(4.12)**	Produced by *Pseudomonas fluorescens*
**7325**	2.22	511.2814	510.2741 (2.17)	$C_{29}H_{86}N_2O_6$ RDB = 12	Maltophilin	Produced from *Stenotrophomonas maltophilia* R3089 (formerly *Pseudomonas maltophilia*
**4766**	2.23	496.3137	495.3064 (1.41)	$C_{24}H_{41}N_5O_6$ RDB = 7	Syringolin A; 2,3-Dihydro	Produced by *Pseudomonas syringae pv. Syringae*
**6832**	2.25	580.3265	579.3193 (-0.59)	$C_{34}H_{45}NO_7$ RDB = 13	Rhizoxin D; O-Demethyl **(4.13)**	Produced by *Burkholderia rhizoxinica* and *Pseudomonas fluorescens* Pf-5
**2899**	2.28	516.2803	515.2731 (-2.58)	$C_{26}H_{37}N_5O_6$ RDB = 11	N-[5-[(1,6-Dihydro-2-hydroxy-5-octanamido-6-oxo-3-pyridyl)imino]-1,2,5,6-	Pigment from *Pseudomonas lemonnieri*

					tetrahydro-2,6-dioxo-3-pyridyl]octanamide	
**2899**	2.28	516.2803	515.2731 (-2.58)	$C_{26}H_{37}N_5O_6$ RDB = 11	Lemonnierin	Produced by *Pseudomonas lemonnieri*
**1146**	2.30	430.1360	429.1257 (0.53)	$C_{19}H_{19}N_5O_7$ RDB = 13	6-(1H-Indol-3-yl)-8-(2,3,4,5-tetrahydroxypentyl)-2,4,7-(1H,3H,8H)-pteridinetrione; D-ribo-form	Isolated from *Pseudomonas ovalis*
**7701**	2.33	596.3223	595.3151 (0.89)	$C_{34}H_{45}NO_8$ RDB = 13	Rhizoxin D; 11R,12R-Epoxide, O-demethyl **(4.14)**	Produced by *Burkholderia rhizoxinica* and *Pseudomonas fluorescens* Pf-5
**6241**	3.00	499.2896	498.2823 (-1.23)	$C_{26}H_{42}O_9$ RDB = 6	Pseudomonic acid A; 4',5'-Didehydro **(4.15)**	Produced by *Pseudomonas fluorescens*
**6375**	3.03	517.3014	516.2941 (1.29)	$C_{26}H_{44}O_{10}$ RDB = 6	Pseudomonic acid A; 5-Hydroxy **(4.16)**	Minor product isolated from *Pseudomonas fluorescens*
**3617**	3.20	627.3500	626.3427 (-0.11)	$C_{32}H_{46}N_6O_7$ RDB = 13	Halotoxin	Produced by *Pseudomonas syringae pv. Mori*
**9567**	3.42	1022.5289	1021.5216 (-.2.28)	$C_{44}H_{71}N_{13}O_{15}$ RDB = 16	Hypeptin	Produced by *Pseudomonas* sp. PB-626
**1903**	4.48	500.3704	499.3631 (1.90)	$C_{26}H_{49}N_3O_6$ RDB = 16	Antibiotic Sch 419559 **(4.17)**	Produced by *Pseudomonas fluorescens*
**5751**	4.51	295.0567	294.0494 (-0.93)	$C_{13}H_{14}N_2O_2S_2$ RDB = 8	Methoxybrassenin B	Stress metabolite isolated from cabbage inoculated with *Pseudomonas cichorii*
**6825**	6.05	496.2437	495.2364 (-1.07)	$C_{27}H_{33}N_3O_6$ RDB = 13	Andrimide; 3-Epimer, 3-hydroxy **(4.18)**	Metabolite of a marine *Pseudomonas fluorescens*
